# Naïve Arrogance and Vulnerability

**DOI:** 10.3201/eid2305.AC2305

**Published:** 2017-05

**Authors:** Byron Breedlove, Paul M. Arguin

**Affiliations:** Centers for Disease Control and Prevention, Atlanta, Georgia, USA

**Keywords:** art science connection, antibiotics, antimicrobial resistance, emerging infectious diseases, art and medicine, about the cover, Florence, Portrait of a Halberdier, Pontormo (Jacopo Carucci), Naïve Arrogance and Vulnerability, public health

**Figure:**
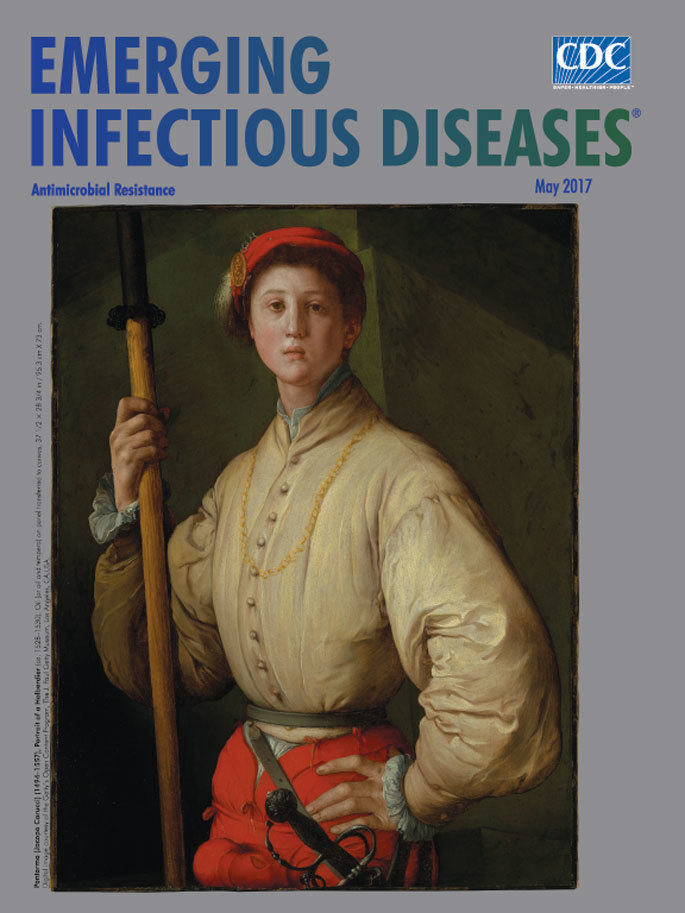
**Pontormo (Jacopo Carucci) (1494–1557), Portrait of a Halberdier (ca. 1528–1530).** **Oil (or Oil and Tempera) on Panel Transferred to Canvas, 37 1/2 in × 28 3/4 in/95.3 cm × 73 cm.** Digital image courtesy of the Getty’s Open Content Program, The J. Paul Getty Museum, Los Angeles, CA, USA.

Florentine painter Jacopo Pontormo, who took his surname from the village of his birth, was considered second only to Michelangelo among his 16th century peers. In Italy, Pontormo was among the first exponents of Mannerism, a style of painting that stressed artifice over realism. The National Gallery of Art offers this view: “Mannerism’s artificiality—its bizarre, sometimes acid color, its illogical compression of space, the elongated proportions and exaggerated anatomy of figures in convoluted, serpentine poses—frequently creates a feeling of anxiety.”

“Portrait of a Halberdier,” this month’s cover image, is considered Pontormo’s masterpiece. The artist completed this painting during the tumultuous 10-month siege of the Florence Republic from October 1529 through August 1530. The J. Paul Getty Museum, which purchased this work in June 1989 notes that as a portraitist, Pontormo “was renowned for his subtle, complex psychological studies; here he conveyed the naïve arrogance and vulnerability of youth.” 

In the painting, Pontormo positions this young foot soldier, holding a combination spear and battle-ax called a halberd, before a bastion. “His direct stare and swaggering pose are strikingly poignant, given the smooth unlined face and slim body that betray him as no more than a teenager,” according to the Getty. The painting conveys ambivalence and tension, for the young man’s display of physical confidence clashes with his anxious countenance and delicate features.

The authors of *Masterpieces of Painting in the J. Paul Getty Museum* state, “Our unbloodied fighter stares into the unknown, his expression suggesting he has just become aware of the myth of the immortality of youth.” This ambivalent message is reinforced by his attire. This young foot soldier casually wears a vivid crimson cap and breech-hose and a fine white padded doublet, replete with a gold chain and cap badge, which depicts Hercules besting the giant Antaeus.

According to art history professor Elizabeth Cropper, this young soldier^1^ displays “none of the brutal qualities that made it worth paying a man to fight, and his elegant costume conveys none of the exaggerated panache of the mismatched gear of a ruffian far from home, a type with which Pontormo was all too familiar in both his life and art.” It is clear that despite having the trappings of strength, this defenseman lacks combat experience and remains quite vulnerable. With this image, Pontormo foreshadows the eventual capture of Florence.

Despite valiant, innovative, and creative tactics, the city of Florence, considered the birthplace of the Renaissance, was outmatched by the large show of force from Spain and the Holy Roman Empire and surrendered after a 10-month siege. Nearly 36,000 of the residents of Florence, about a third of the city’s population, died during the siege, including many young people pressed to defend the city. Florence’s citizens sustained other losses as well, including the ruin of their various export businesses and confiscation of their wealth and possessions.

It is tempting to view the issue of antimicrobial resistance—the ability of a microorganism to stop an antimicrobial from working against it—in terms of a siege. Standard treatments become ineffective over time, infections persist, and organisms mutate and propagate. Antimicrobials are armed with the ability to interfere with bacterial cell wall synthesis, to prevent pathogenic protein formation, or to alter bacterial metabolic processes. Those mechanisms of action are akin to the armaments of our stalwart halberdier.

However, whether by the naïve arrogance of overuse and misuse or by evolutionary bad luck, such weapons in the fight against infections are losing their effectiveness, potentially leaving us as vulnerable as Florence. To withstand the siege of antibiotic resistance, innovative strategies for preserving the effectiveness of our existing antimicrobials and for developing new drugs with novel mechanisms of action are needed.

A besieged city that finds its resources dwindling, allies absent, and enemies undeterred will eventually fall. In public health, putting on a brave face will not save the day. Public health must have the capacity to detect, respond to, and prevent antibiotic-resistance threats from a host of bacteria, fungi, viruses, parasites, and other microorganisms. Mounting effective responses across healthcare settings and communities across the world requires investments, infrastructure, and collaboration, including a One Health strategy, which involves partners representing the human, animal, and environmental determinants of health.

## Footnotes

1 A long-running debate over the identity of the youth who posed for this portrait lies outside the scope of this essay. Cropper argues that he is a young man named Francesco Guardi in *Pontormo: Portrait of a Halberdier*.
